# p38α Negatively Regulates Survival and Malignant Selection of Transformed Bronchioalveolar Stem Cells

**DOI:** 10.1371/journal.pone.0078911

**Published:** 2013-11-12

**Authors:** Edwige Voisset, Feride Oeztuerk-Winder, Edgar-Josue Ruiz, Juan-Jose Ventura

**Affiliations:** CSCR (Wellcome Trust Centre for Stem Cell Research), Cambridge, United Kingdom; University of Colorado, Denver, United States of America

## Abstract

Lung cancer is the cause of most cancer-related deaths in the Western world. Non-small cell lung cancer accounts for almost 80% of all lung cancers, and 50% of this type are adenocarcinomas. The cellular and molecular origin of this type of lung cancer remains elusive and the mechanisms are poorly known. It is known that K-Ras mutations appear in 25–30% of lung adenocarcinomas and it is the best known single mutation that can be related to lung cancers. Recently, it has been suggested that a putative population of mouse bronchioalveolar stem cells could be considered as the cell of origin of adenocarcinomas. These cells are expanded in the early stages of lung tumorigenesis. We have isolated a population of mouse bronchioalveolar stem cells and induced their transformation by oncogenic K-RasG12. Different approaches have shown that an intracellular network linking the p38α MAPK and the PI3K-Pdk1 pathways is involved in regulating the survival and malignant progression of the transformed cells. Absence of p38α catalytic activity leads to further Pdk1 activation (independent of Akt and Erk activity), enhancing the survival and proliferation of the more malignant lung cancer cells. This specifically selects high Sca-1/Sox9 cells that harbour a stronger colonizing potential, as they maintain their capacity to produce secondary tumors after serial transplantations.

## Introduction

Lung cancer is the main cause of all cancer deaths in the world [1]. However, after many years of research, very little is known about the cellular and molecular mechanisms involved in this type of cancer. The existence of a population of lung cells that could be the source of the major types of lung cancers has been studied in recent years, but still remains elusive and very controversial. Recently, different studies in animal models have suggested the existence of a population of bronchioalveolar stem cells that could act as the cell of origin [3], [4]. These cells are expanded in the first stages of lung tumorigenesis induced by oncogenes. However, the mechanisms that promote these cells to transformation, and their progression to malignant stages are still poorly understood [5].

Using a mouse model of lung cancer induced by oncogenic K-RasG12, we have previously determined the role of the p38α pathway opposing the initiation and progression of the tumorigenic process [4]. This kinase promotes differentiation factors that act as negative regulators of tumorigenesis (e.g. C/EBPα), while reducing proliferation of lung stem cells. Human lung tumours show a selection of cells with low levels of p38α protein [4]. To further study the progression of lung cancer and define the mediators of that repressive role by p38α, we have isolated putative stem cells from mouse lungs with a specific expression profile (Sca-1^+^/E-Cadherin^+^/CD31^−^/CD34^−^/CD45^−^/CD73^−^). These cells can be indefinitely expanded in culture and transformed by expression of a K-RasG12 mutant.

In this manuscript we have studied, *in vitro* and *in vivo*, the process of transformation of the lung stem cells and the mechanisms involved in the selection of tumour cells with low levels of p38α, and the functional advantages that low p38 activity provides to the progression of lung cancer to more malignant stages.

## Materials and Methods

### Cell Isolation and Cell Culture

Mouse lung tissues from wild-type or *LSL-K-ras^G12D^ –TetO-sftpc-Cre* C57BL/6 mice were disassociated by finely mincing with a razor blade and by incubating in Dulbecco’s modified Eagle’s medium (DMEM, PAA) containing collagenase (3 mg/mL, Worthington Biochemical) for 30 minutes at 37°C in a shaking incubator. The resulting cell suspension was submitted to a series of three filtrations (two through a 70-µm strainer and the last one through a 40-µm strainer). Then cells were FACS sorted to select Sca1^+^/E-Cadherin^+^/CD31^−^/CD34^−^/CD45^−^/CD73^−^ cells. Sorted Sca1^+^ cells were used for SC injections or re-suspended in DMEM supplemented with 10% fetal bovine serum (FBS, PAA), insulin (50 ug/mL, Peprotech), EGF (10 ng/mL, Peprotech), FGF2 (10 ng/mL, Peprotech) and 1% penicillin/streptomycin (PAA). After two days, the medium was changed for serum free DMEM/F12 (ratio 1∶1) supplemented with 1% N2, 2% B27 serum (Gibco), EGF (10 ng/mL) and FGF2 (10 ng/mL). Cells were seeded in normal culturing plates.

The medium was changed every two days and once a week an accutase (PAA) treatment was perform to disassociate spheres. Cells were grown in a 7% CO_2_ incubator at 37°C.

Mouse K-Ras tumor cells derived from *LSL-K-ras^G12D^ –TetO-spc-Cre* lung adenocarcinomas were isolated from 15 week tumors following the above mentioned protocol and then sorted for Sca1 and used for SC injections.

### Retroviral Infection

Retroviruses were produced according to a protocol from the Weinberg’s lab (Addgene). One of the retroviral plasmid vectors (pBabe-K-RasWT-GFP, pBabe-KRasG12V-GFP and pBabe-p38AGF) and the packaging plasmids (M57 and pMD.G2) were co-transfected using CaPO_4_ precipitation (Invitrogen) into the packaging cell line 293T. Viral supernatants were collected 48 hours later, filtered through 0.45-µm filters and concentrated using Spin-X® UF concentrators (100 KD MWCO, Corning). After selection by cell sorting on GFP positive cells, K-RasWT and G12V cells have been infected with pBabe-p38AGF. 48 hours after infection, 5 ug/mL of puromycin were added to the medium.

### Flow Cytometry Analysis

All staining were performed in PBS (PAA) with 1% BSA (Sigma). Antibodies used are listed below: Sca-1 (PE-conjugated) and E-Cadherin (Alexa 647-conjugated) purchased from Biolegend; CD34 (FITC-conjugated), CD31 (FITC-conjugated) and CD45 (FITC-conjugated) from BD Pharmingen and CD44 (PerCP-Cy5.5-conjugated) from eBiosciences. Flow cytometry analyses were performed on a MoFlo cytometer (Dako). All data were analysed with FlowJo software (Tree Star).

### Mouse Experiments

All animal experimentations were performed in accordance with the terms of UK Home Office guidelines. The home office project license number under which these experiments were conducted is PPL 80/2188. CD1 nude male mice were obtained from Charles Rivers Laboratories.

Ten week old mice received subcutaneous injection in each flank of 10^4^ cells. Animals were monitored daily. Tumour volume was measured using an electronic digital calliper and calculated as an ellipsoid [6], [7].

Subcutaneous tumours formed after injection of cells were removed. Pictures were taken under microdissection microscope (brightfield and GFP). A part of each tumours were fixed in 4% paraformaldehyde for 24 hours at 4°C, then placed in 30% sucrose overnight prior to embedding the tissue in OCT (Sakura). Slides were stained in hematoxylin and eosin (H&E Dako) or with Ki67 (Vector Labs) according to the manufacturer’s instructions. Images were acquired by using a Zeiss Apotome microscope.

Each tumour was disassociated to obtain single-cell suspension. For that, tumour tissues were disassociated mechanically and enzymatically. Tumors were minced with a razor blade and incubated in accutase at 37°C for 10 minutes. The tissues were further dissociated by pipette trituration and filtered through a 70-µm strainer. Then cells were re-suspended in DMEM/F12 (ratio 1∶1) supplemented with 1% N2, 2% B27 serum, EGF (10 ng/mL) and FGF2 (10 ng/mL).

### Total RNA Isolation, Reverse Transcription and PCR

Total RNAs were extracted using TRIzol (Invitrogen) according to manufacturer’s instructions. The RNA solutions were treated with RNase-free DNase I (Promega) to remove trace of genomic DNA contamination. Total RNAs (1 ug) of each sample were reverse transcribed into cDNAs using the iScript cDNA synthesis kit (Bio-Rad) containing oligo(dT) according to the manufacturer’s instructions. Reactions were carried out using a Mastercycler Gradient (Eppendorf) under the parameters and primers described as following: 94°C for 30 s, 49°C for 30 s, and 72°C for 30 s for 30 cycles with sense 5′-CTTTATCCAGCCCTCAC-3′ and anti-sense 5′-ACCCTAACTGACACACATTCC-3′ for p38AGF; 94°C for 30 s, 53.2°C for 30 s, and 72°C for 30 s for 30 cycles with sense 5′-CTTGTGGTGGTTGGAGCTGTA-3′ and anti-sense 5′-CTCCCCAGTTCTCATGTACTGG-3′ for K-RasG12V.

### Quantitative Real-Time PCR

cDNA products were used for further performance of the quantitative real-time PCR (qPCR) under the conditions and primers described as followings: initial denaturation at 95°C for 20 s followed by 40 cycles with denaturation at 95°C for 3 s, annealing at 60°C for 30 s and elongation at 68°C for 20 s. The primers used in the experiments were sense 5′-AGTACCCGCATCTGCACAAC-3′ and anti-sense 5′-ACGAAGGGTCTCTTCTCGCT-3′ for sox9;; sense 5′-AGGTCGGTGTGAACGGATTTG-3′ and anti-sense 5′-TGTAGACCATGTAGTTGAGGTCA-3′ for gapdh; sense 5′-GGCTGTATTCCCCTCCATCG-3′ and anti-sense 5′-CCAGTTGGTAACAATGCCATGT-3′ for beta-actin. All samples were analyzed in triplicate by Fast SYBR green master mix (Applied Biosystems) using a Mastercycler ep realplex^2^ real-time PCR machine (Eppendorf). Relative gene quantification was performed using the ΔΔCT method.

### Viability, Cell Cycle and Cell Death Analysis

Cell viability assays were done with a Vi-CELL XR cell viability analyzer with Vi-CELL XR 2.03 software (Beckman Coulter). The small chemical inhibitors used are Akt Inhibitor 1/2 (AI, Sigma) at 10 µM, PI3Kinase Inhibitor (LY294002 or LY, Sigma) at 20 µM, and DMSO (Sigma) as control.

Cells were incubated with 10 µM EdU at 37°C for 120 minutes. The cells were then accutase treated, and fixed in 4% paraformaldehyde. EdU incorporation was determined with the Click-iT EdU Alexa Fluor 647 flow cytometry assay kit (Molecular Probes) according to the manufacturer’s instructions. For each population, a minimum of 10^4^ cells was counted. Cells were analysed on Fortessa flow cytometer (Becton Dickinson) using FACSDiva software and data analyses were performed with FlowJo software.

Apoptosis was determined by Annexin V-APC/Dapi staining using Apoptosis Detection Kit (BD Pharmingen). These assays were performed according to manufacturer’s instructions. For each population, a minimum of 10^4^ cells was counted. Cells were analysed on Fortessa flow cytometer (Becton Dickinson) using FACSDiva software and data analyses were performed with FlowJo software.

### Anchorage-Independent Growth Assays

5000 cells/well were seeded in six-well plates within a top layer of 0.3% agar and a bottom layer of 0.6% agar. After the top layers were solidified, 1 mL of media was added. During the incubation, the plates were checked every day and media replenished each 3 days. Plates were stained with 0.005% crystal violet for 1 hour, and the colonies were counted.

### Western-Blotting

Cell lysates were prepared with RIPA buffer (50 mM Tris-HCl, pH7.5, 150 mM NaCl, 1.0% Triton X-100, 0.1% SDS, 1% sodium deoxycholate, 1 mM pervanadate, protease inhibitor cocktail tablet (Roche Diagnostics Ltd)) on ice for 1 hour. 30 µg of the total protein was applied to a SDS-PAGE, transferred to PVDF membrane (Millipore) and blocked for 1 hour in blocking buffer (5% BSA). Blots were incubated overnight at 4°C with primary antibodies in blocking buffer. The primary antibodies used were: anti-Cyclin D1, purchased from Santa Cruz Biotechnology; anti-p38, anti-Akt, anti-pAkt Ser473, anti-pPdk1 Ser241, anti-Pten, anti-pPten Ser380 from Cell Signaling Technology; anti-Sox9 from Millipore, anti-pERK1/2 from Promega and anti-Tubulin from Sigma. IR-dye-labelled secondary antibodies (LI-COR) were applied at 1∶15000 for 1 hour. All blots were imaged using the Odyssey infrared scanner (LI-COR). Each western-blot was repeated at least three times.

## Results

### Molecular and Functional Characterization of *in vitro* Expanded K-Ras-transformed Bronchioalveolar Stem Cells

**Figure 1:**
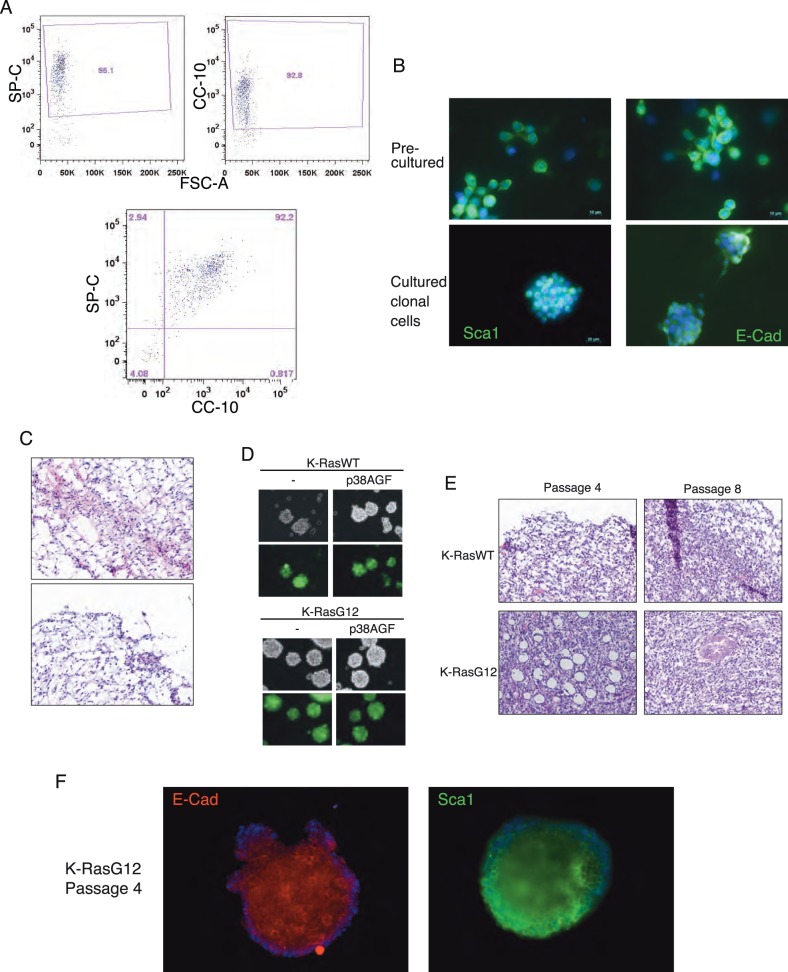
*In vitro* and *in vivo* characterization of bronchioalveolar and K-Ras WT or G12 expressing lung stem/progenitor cells. Mouse lung bronchioalveolar stem cells were isolated negatively sorting for hematopoietic, endothelial and mesenchymal markers and positive for E-Cad/Sca1. (A) Immunofluorescence staining shows that freshly sorted (upper), or clonally derived cells cultured for 30 passages (lower) express Sca1 and E-Cadherin. (B) Freshly sorted (upper) or clonally cultured cells (lower) have the potential to generate bronchioalveolar tissue in kidney capsule transplants. (C) Clonally culture stem/progenitor cells (passage 3) were transfected with K-RasWT, K-RasG12 and/or p38AGF (dominant negative) vectors. (D) H&E staining of kidney capsule outgrowths produced by K-RasWT or K-RasG12 cells at different passages. (E) K-RasG12 infected cells maintain E-Cad and Sca1 expression after passage 4.

As previously published [4], putative lung stem cells were isolated from mouse bronchioalveolar epithelium using a negative (CD31^−^/CD34^−^/CD45^−^/CD73^−^) and a positive (E-Cadherin^+^/Sca-1^+^) selection (Fig. 1A). Clonally derived cells can be cultured and maintain the epithelial (E-Cad) and stem (Sca1) cell markers (Fig. 1A). The cultured cells retain a similar differentiation potential as the freshly isolated cells as shown by kidney capsule transplants (Fig. 1B). These cells grow in spheres in culture and single cell clones can be indefinitely expanded while expressing lung specific and stem cell markers (Fig. S1A and S1B). To study oncogenic transformation, clonally expanded cells were stably transfected with retroviral vectors expressing K-RasWT or mutant K-RasG12. In addition, the cells were co-infected with retroviral vectors expressing a dominant negative mutant form of p38α (p38AGF) that inhibits the pathway (Fig. 1C and Fig. S1C). K-RasWT infected cells retain the potential to generate a bronchioalveolar epithelium in kidney capsule transplants after 8 passages, but the transformed K-RasG12 infected cells give rise to an undifferentiated tissue with tumorigenic features (Fig. 1D). The K-RasG12 infected cells still express the selection markers (E-Cad/Sca1) at passage 4 (Fig. 1E).

**Figure 2:**
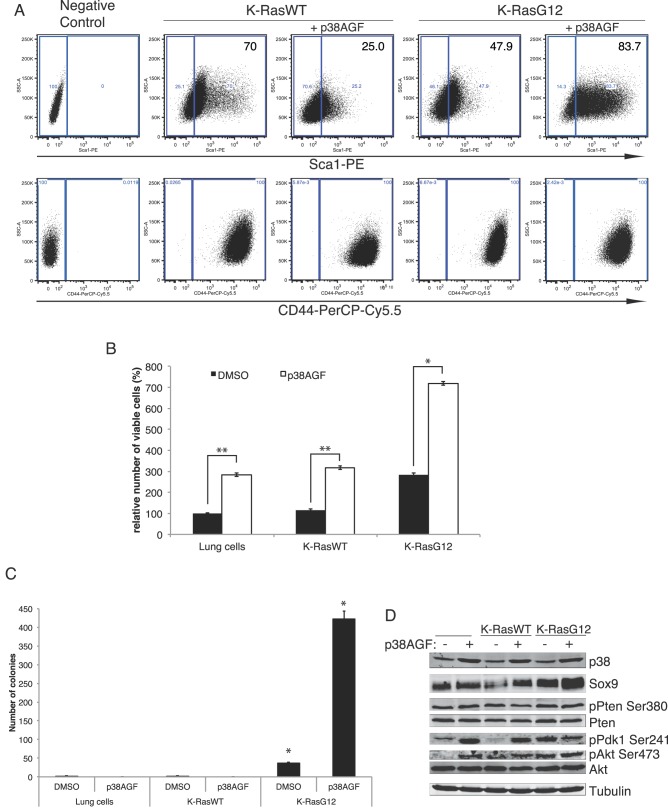
Molecular and functional characterization of K-RasWT and K-RasG12 infected bronchioalveolar stem/progenitor cells. (A) Flow cytometry plots showing the expression of the stem cell markers Sca-1 (upper) and CD44 (lower) in cells stably expressing K-RasWT, K-RasG12 and the dominant-negative p38AGF 4–6 weeks after the infection with the corresponding constructs. The plots show a representative experiment of 5 different made in triplicates. K-RasWT, K-RasG12 and K-RasG12+AGF, p≤0.01; K-RasWT+AGF, p≤0.05. (B) p38 inhibition collaborates with K-RasG12 transformation to increase cell viability of lung stem/progenitor cells. (C) Absence of p38 activity specifically increases soft agar colony forming potential of transformed K-RasG12 lung stem/progenitor cells. (D) p38 deficiency constitutively activates the PI3K pathway, increasing P-Pdk1 and P-Akt but not P-Pten levels.

There is an *in vitro* differential selection of Sca-1 cells; whereas the WT K-Ras cells maintain higher levels of putative progenitor/stem cells (Sca-1^+^), the oncogenic-induced transformation lowers the Sca-1^+^ population. However, inhibition of the p38α pathway produces the converse selection, reducing Sca-1^+^ WT cells and increasing that population of transformed K-RasG12 cells (Fig. 2A). The expression of the cell adhesion molecule CD44 recently described as able to promote Ras-mediated MAPK signaling [8] is not affected, although is higher in the K-RasG12 cells (Fig. 2A).

Deficiency of p38 signal increases the survival of WT and transformed cells, but is significantly higher in the K-RasG12 cells (Fig. 2B). The p38-dependent increased survival is independent of canonical apoptosis (Fig. S1D) and proliferation induced by K-RasG12 (Fig. S1E). However, lack of p38 signalling specifically promotes anchoring-independent growth in soft agar (a hallmark of cellular transformation), of oncogenic but not of WT cells (Fig. 2C). Correlating to the transforming capacity, absence of p38 activity induces an upregulation of the expression of the stem and lung cancer marker Sox9 [9], [10] in K-RasG12 cells (Fig. 2D and Fig. S1F) supporting its potential as a therapeutic target.

### p38 Crosstalk with PI3K-Pdk1 is Involved in Stem Cell Selection

**Figure 3:**
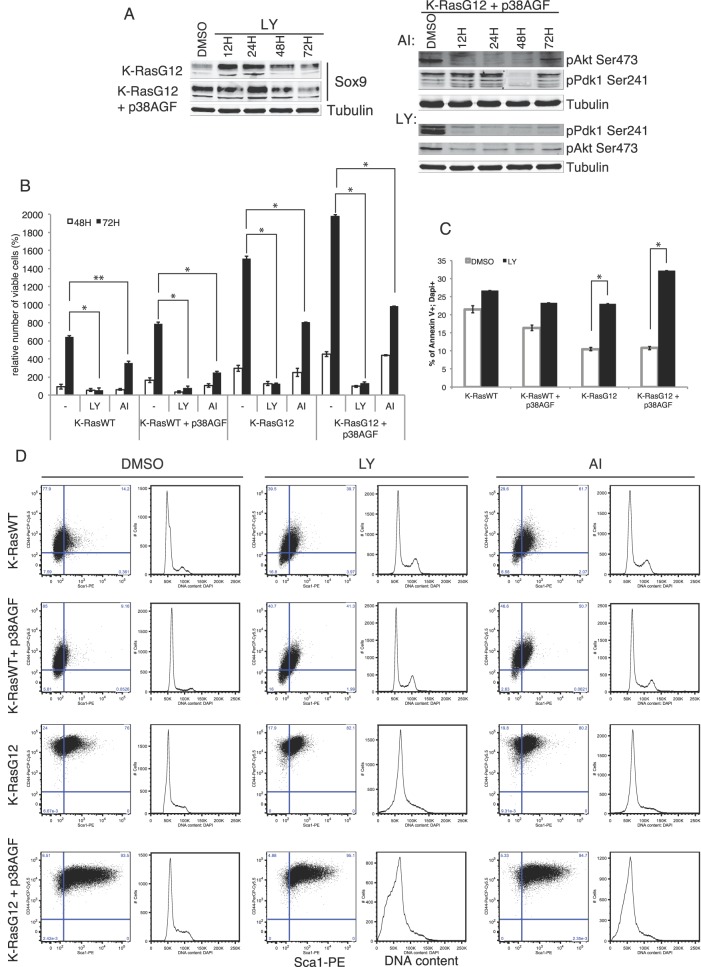
PI3K-Pdk1 dependent survival of K-RasG12 transformed lung stem/progenitor cells selects for CD44^+^/Sca-1^+^ populations. (A) Protein levels of the stem cell marker Sox9 are reduced by PI3K inhibition in K-RasG12 transformed lung stem/progenitor cells. PI3K inhibition, but not specific Akt inhibition, reduces the Pdk1 constitutive activation in K-RasG12 cells deficient in p38 signalling. (B) Survival levels of K-RasWT and/or K-RasG12 lung stem cells with or without active p38. (C) PI3K inhibition increases classic apoptosis of oncogene transformed lung stem/progenitor but not K-RasWT cells. (D) Absence of p38 signalling selects for high CD44^+^/Sca-1^+^ expressing K-RasG12 cells. PI3K inhibition significantly and specifically increased cell death of p38-deficient oncogenic cells. The plots are from a representative experiment of 7 different made in triplicates. P value: p≤0.01 to p≤0.03.

It has been reported by others and us that p38α signalling deficiency results in constitutive activation of other kinase pathways such as JNK, ERK or AKT [4]. To test the possible mediation of any of these pathways in the functional role of p38α in lung stem cell transformation, we used different small chemical inhibitors. The ERK and PI3K pathways are major mediators of oncogenic K-Ras induction of transformation. A chemical inhibitor of PI3K was able to repress Sox9 protein levels in transformed cells deficient or not in p38 signal (Fig. 3A). Also the inhibition of PI3K activity, but not Akt, efficiently reduced Akt and Pdk1 constitutive activation in absence of p38 signalling (Fig. 3A) when Akt inhibition itself only marginally reduced cell viability (Fig. 3B). However, inhibition of PI3K almost completely abrogated cell viability (Fig. 3B), in part due to an increase in canonical apoptosis (Fig. 3C). Therefore, Pdk1 but not Akt is mediating the increased survival of oncogenic transformed lung stem cells. In addition, the mostly Sca-1^+^/CD44^+^ p38-deficient transformed lung stem cells are more sensitive to PI3K induced cell death than the WT or K-RasG12 cells with normal p38 signalling (Fig. 3D). Interestingly chemical inhibition of ERK did not affect Pdk1 activation (data not shown) weakening the hypothesis of a potential cross-talk between these two related pathways.

### Sca-1 is a Marker of Tumorigenic Potential for K-RasG12 Transformed Lung Progenitor/Stem Cells

**Figure 4:**
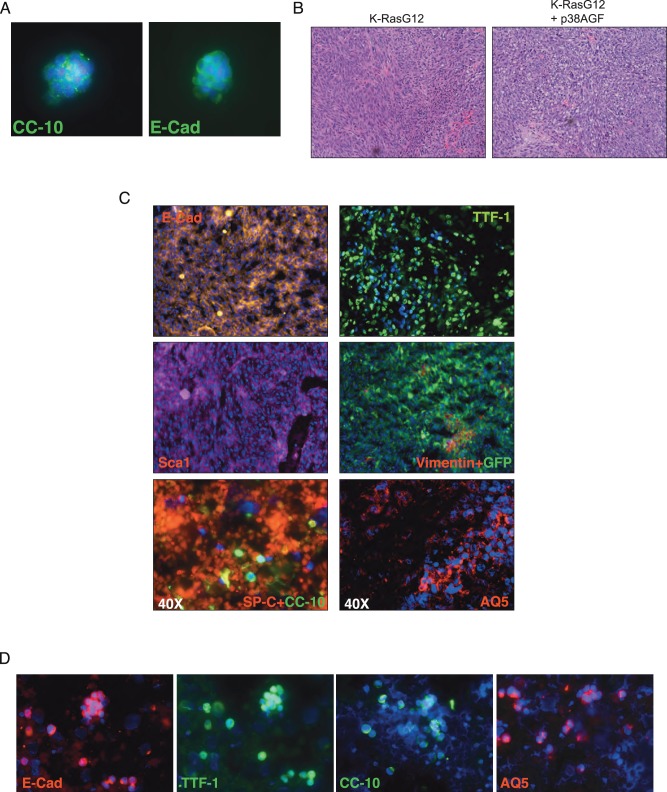
Cellular characterization of Subcutaneous Tumors induced by K-RasG12 transplanted cells. (A) Transformed K-RasG12 (+/−p38AGF) cells (passage 4) used for subcutaneous (SC) injections express epithelial and lung specific markers. (B) H&E staining of SC tumours induced by K-RasG12 (+/−p38AGF) cells. (C) Immunostaining of K-RasG12-induced SC tumors with epithelial (E-Cad), lung (TTF-1, SP-C, CC-10, AQ5), stem (Sca1) or mesenchymal (Vimentin) markers. (D) Cells isolated from K-RasG12-induced SC tumors express different levels of epithelial and lung specific markers.

Deficient p38α signalling selected K-RasG12 transformed cells for a more stem cell profile with higher *in vitro* transforming potential. We tested the *in vivo* tumorigenic capacity of these cells using subcutaneous (SC) injections in nude mice. For the injections we used clonally derived cells expressing K-RasG12 at passage 4 after infection. These cells maintained the expression of lung and epithelial markers (Fig. 4A). Injected K-RasG12 and K-RasG12+p38AGF cells (10^4^ cells) formed subcutaneous tumors (Fig. 4B). Neither non-infected or K-RasWT cells were able to grow when SC injected (data not shown). K-RasG12 SC tumors were formed by cells expressing epithelial (E-Cad), stem (Sca1) and lung specific (TTF-1, SP-C, CC-10, AQ5) markers, but not the mesenchymal marker vimentin (Fig. 4C). Cells isolated from SC tumors confirmed different expression levels of epithelial and lung specific markers (Fig. 4D).

**Figure 5:**
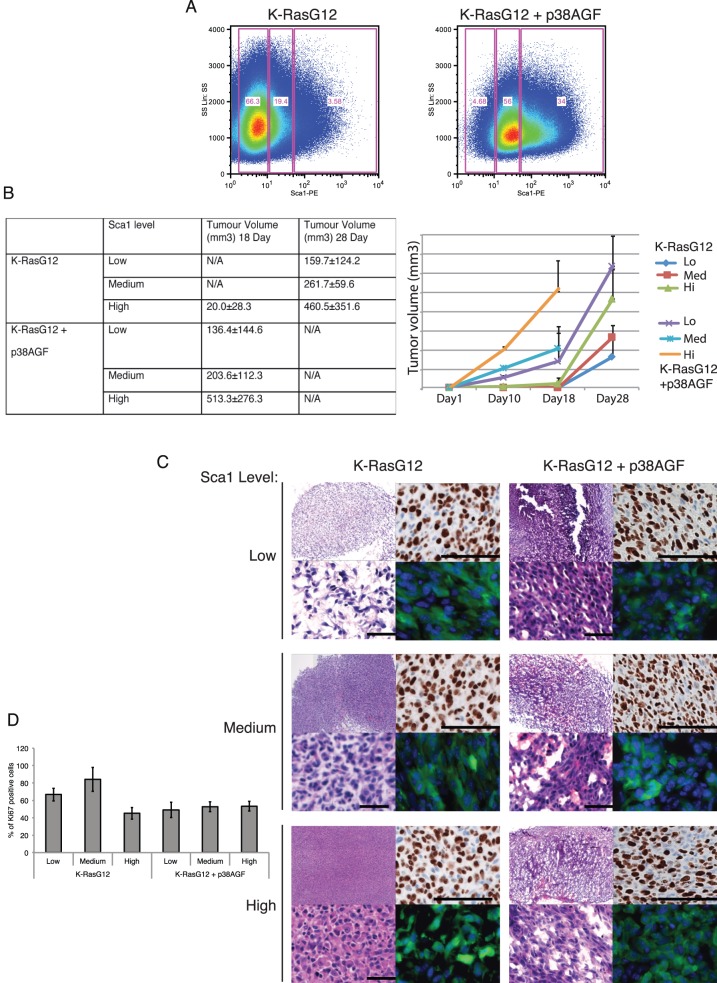
Sca-1 level determine a differential tumorigenic potential of K-RasG12 transformed lung stem cells. (A) Distinct Sca-1 populations in K-RasG12 transformed stem/progenitor cells based on p38 signalling. (B) Lack of p38 signalling and higher Sca-1 levels in K-RasG12 cells correlate with increased potential to produce subcutaneous tumours. One-Way ANOVA test p≤0.05. (C) H&E images of subcutaneous tumors induced by different populations of K-RasG12 or K-RasG12+AGF with low, medium or high levels of Sca-1. Right panels show Ki67 expression and GFP detection of injected cells (scale bar 50 µM). (D) Bar graph showing the Ki67 expression of Sca-1 populations of K-RasG12 WT or AGF. A representative graph of 3 to 5 independent experiments is shown, ±SD.

Based on different levels of Sca-1 expression, we sorted K-RasG12 or K-RasG12+p38AGF (K-RasG12+AGF) populations with low, medium or high level of expression of that stem cell marker (Fig. 5A). Cells from every population (10^4^ cells) were subcutaneously injected in nude mice. As a control, K-RasWT cells were injected but did not induce any tumors. Mice were sacrificed for humanitarian reasons when the animals showed distressful behaviour. After 18 days, most K-RasG12+AGF injected animals had to be sacrificed. At this time, it was obvious that the size of the tumors correlated with higher Sca-1 expression cells (Fig. 5B). Of the K-RasG12, only the high Sca-1 cells produced tumors at day 18, but not the low and medium Sca-1 expressing cells. Only after 28 days all K-RasG12 populations generated tumors, and the size also correlated with the level of Sca-1 expression (Fig. 5B). The morphology of the tumors was also distinct based on the lack of p38 activity and Sca-1 expression. All p38-deficient cells showed loose and not well encapsulated tumours with high vascularisation (Fig. 5C right). On the other hand, the p38 active tumors were well-delimited and less vascularised (Fig. 5C left). Cells with high Sca-1 expression showed a poorly differentiated morphology, and that was enhanced in the tumors lacking p38 signal. Interestingly, the percentage of Ki67 proliferating cells is higher in the tumors with p38 activity and low level of Sca-1 expression (Fig. 5D).

### p38 Opposes the Tumorigenic Selection for More Stem Cell-like Aggressive Tumor Cells

**Figure 6:**
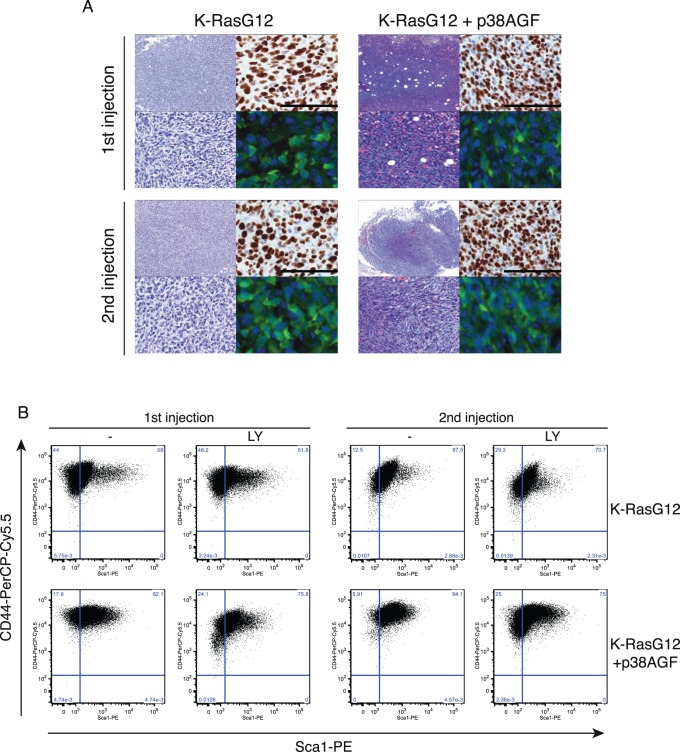
Tumorigenic progression selects for CD44^+^/Sca-1^+^ cells and is promoted in absence of p38 signalling. (A) Tumor morphology and Ki67 expression in serial subcutaneous injections of K-RasG12 and K-RasG12+AGF cells (scale bar 50 µM). n = 10, 1^st^ transplant; n = 8, 2^nd^ transplant (B) Flow cytometry plots showing CD44 and/or Sca-1 expression in K-RasG12 or K-RasG12+AGF isolated from sequential tumours and the same populations after PI3K inhibition for 72 hours. 1^st^ transplant, n = 10, p≤0.01; 2^nd^ transplant, n = 8, p≤0.05.

The more invasive and malignant properties of the transformed lung stem cells lacking p38 activity were obvious after serial tumor inductions (Fig. 6A and Fig. S2A). Transformed cells expressing a GFP reporter (green) were isolated from subcutaneous tumor, sorted and directly injected subcutaneously in nude mice. Serial tumors showed again more vascularized and less differentiated tissue in p38-deficient cells (Fig. 6A). Cells isolated from tumors were used for *in vitro* soft agar assays. K-RasG12 cells increased their potential to form colonies after serial tumorigenesis, but that was especially obvious for the cells lacking p38 signalling (Fig. S2A). On the other hand, K-RasWT cells failed to induce tumors or produce colonies in soft agar (Fig. 2C and data not shown). The increase in transforming potential gained by K-Ras oncogenic cells in serial injections correlates with higher expression of the stem cell marker Sox9 whereas there is no modification of the CyclinD1 expression level (Fig. S2B). Oncogenic K-RasG12 cells lacking p38 signalling were selected for cells with higher levels of stem cell markers CD44 and Sca-1 expression than their counterparts with a normal p38 activity (Fig. 6B). That selection is prevented by inhibition of PI3K and especially is active in reducing the CD44^+^/Sca-1^+^ population of p38-deficient K-RasG12 cells from 1^st^ or 2^nd^ induced tumorigenesis. Cells in 2^nd^ injection SC tumors did not express non-epithelial markers (Fig. S2C) but still expressed the lung specific marker SP-C (Fig. S2D). Furthermore, only the K-RasG12 cells from secondary tumors are sensitive to PI3K inhibition reducing the selection to Sca-1 cells (Fig. 6B). Moreover, these two transformed cell populations are able to colonise lungs and to form lung tumors and once again p38 inhibition increases the tumorigenic potential in the lung of K-RasG12 as shown in tail vein injections (Fig. S2F).

**Figure 7:**
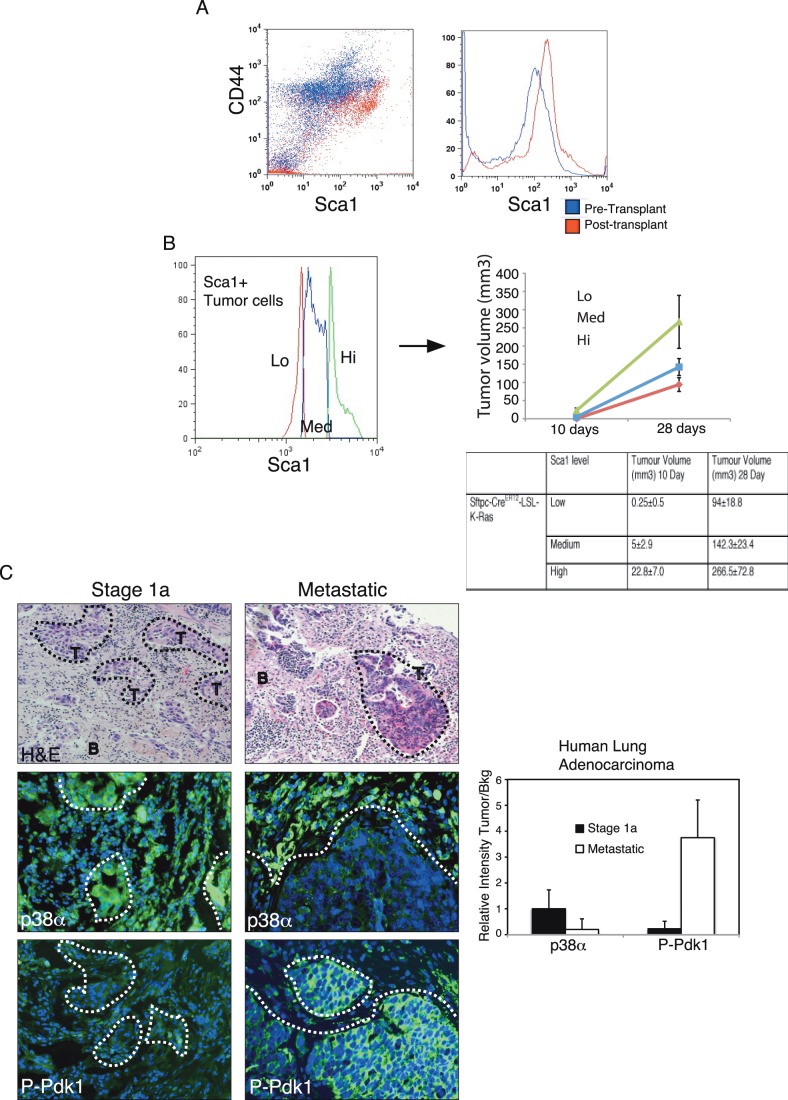
Mouse and Human Adenocarcinomas show a correlate Sca1 selection to p38α deficiency, increased P-Pdk1 and malignant progression. (A) Flow cytometry analysis of CD44 and Sca1 expression of tumor mouse LSL-K-Ras cells before (blue) and after (red) SC induced tumors. (B) Mouse LSL-K-RasG12 (Sca1+) tumor cells were sorted for different levels of Sca1 expression and SC injected in nude mice (10^4^ cells). The graph depicts the evolution of tumor volumes show in the below table at 10 or 28 days after transplantation. One-Way ANOVA test p≤0.05. (C) Immunostaining of tissue samples showing H&E and the expression of p38α (centre) and P-Pdk1 (low) in K-Ras mutant early (stage 1) or late (metastatic) human lung adenocarcinomas. Graph shows the relative intensity of p38α and P-Pdk1 expression in the tumor (T) areas versus the expression in the background (B) tissue. Stage 1, n:13; Metastatic, n:9, are depicted.

Sca1-dependent selection of K-Ras tumor cells during tumorigenic progression was confirmed using tumor cells from a *LSL-K-ras^G12D^ –TetO-sftpc-Cre* mouse model of lung adenocarcinoma [11]. Cells from 15-week old tumors were sorted for Sca-1 expression and directly used for SC injections. Cells isolated from 20 days old SC tumors express higher levels of Sca1 than the LSL-K-Ras tumor cells used for the injections (Fig. 7A). LSL-K-RasG12 (15 week) tumor cells were also differentially sorted in low, medium or high Sca1 levels and injected SC in nude mice. Sca1 expressing levels determined a differential potential to induce tumors in time and size (Fig. 7B), confirming the results observed with the transformed cells in culture.

Selection of p38α deficient cells was previously shown in human lung cancers [4]. Histological analysis of samples from human adenocarcinomas carrying mutant K-Ras12 at different stages of cancer progression, have shown a correlation between loss of p38α, activation of Pdk1 (pPdk1) within the tumors and progression of human lung adenocarcinomas into malignant stages (Fig. 7C). Thus, we confirmed that pPdk1-dependent selection of more malignant Sca-1 cells is a common mechanism during adenocarcinoma progression in mouse and human lung cancers.

## Discussion

The cellular progression of cancer cells into malignant stages is still poorly understood [12]. Loss of differentiation markers and morphology are some of the hallmarks of lung cancer development (e.g. from adenomas to adenocarcinomas) [11]. It is known that accumulation of mutations happens during the malignant progression [12]. However, most of the mutations in lung cancer either play a small role or none at all [13]. Oncogenic (G12) K-Ras is present in an important percentage of lung cancers and especially in adenocarcinomas [1]. Some of the pathways involved in K-Ras signalling are well-known and drugs directed against molecules at different levels of these pathways (e.g. ERK, PI3K, AKT or mTOR) are already being used in the clinic or undergoing clinical trials [2]. Nevertheless, the efficiency of these drugs has been very limited and has not fulfilled the expectations. One of the problems in cancer therapy is the existence of small populations of cells resistant to the treatment that relapses as more virulent and malignant than the previous tumor [14].

We have studied the malignant progression in mouse lung cancer using a population of cells that has previously proposed to be a putative cell of origin of cancer [3], [4]. Lung cells were transformed inducing the expression of a mutant K-RasG12 [15]. These transformed cells are able to colonize the lungs produced subcutaneous tumors, harbouring the potential to induce sencondary tumors after transplantation. Our data supports the role of the p38 MAPK pathway opposing undifferentiation promoted by transformation [4]. Deficient p38 signalling is accompanied by constitutive activation of PI3K and ERK pathways [4]. It is the PI3K-Pdk1 pathway, but not Akt or Erk what increases the survival of more stem-like cells with higher levels of the stem and cancer markers Sca-1, CD44 and Sox9, in absence of p38 activity. Previous studies suggest Sox9 and Sca1 as potential therapeutic target and marker of tumorogenicity respectively. Interestingly, in our context, expression of these proteins are also related to the severity/efficiency of the oncogene K-Ras (+/− p38 activity). Inhibition of the PI3K-Pdk1 axis specially affects the high CD44^+^/Sca-1^+^ population, increasing cell death and reducing cell proliferation. This is important as higher Sca-1 expression cells have more potential to produce colonies in soft agar and faster and bigger subcutaneous or lung tumors. High Sca-1 tumour cells are more tumorigenic and they are selected in secondary and later tumors.

We have defined a mechanism that links the known K-Ras pathway, PI3K-Pdk1 with the previously observed low p38 activity found in lung adenocarcinomas. Reduced p38 activity allows more PI3K-Pdk1 signalling, that selects more malignant cells, with a less differentiated stem-like profile, with expression of Sca-1/CD44/Sox9 markers. The confirmation of a similar mechanism in human lung adenocarcinomas provides a further advance in the search of putative new targets for therapy and detection of more malignant cells.

## Supporting Information

Figure S1 **In vitro culture and characterization of K-Ras transformed cells.** (A) Immunofluorescence staining of K-Ras12 transformed Sca1 sorted cell spheres in culture showing the expression of epithelial, stem and lung specific markers. (B) Graph shows qPCR relative mRNA expression of lung specific markers in freshly isolated, cultured (passage 30) or K-RasG12 infected (passage 4). (C) Lung stem cells transfected with retrovial vectors expressing K-RasWT or K-RasG12V. The PCR shows the expression of p38AGF (dominant negative), or oncogenic K-RasG12V. (D) Apoptosis of the cell types based on Anexin detection by flow cytometry. (E) Cell cycle distribution of the different cell types. (F) Relative mRNA expression of Sox9 in cells with or without p38 activity.
(PDF)

Figure S2 ***In vitro*** **and** ***in vivo*** **characterization of the functional and molecular properties of the transformed stem cells.** (A) Colony formation in soft agar using K-Ras transformed lung stem cells in culture or isolated from serial subcutaneous tumours. Values are depicted as mean ± standard error of the mean (SEM) from four different experiments. (B) Sox9 and Cyclin D1 protein expression in K-RasWT or K-RasG12 with or without p38 activity in culture or cells isolated from serial tumours. (C) Injected GFP cells in SC tumors do not express the non-epithelial marker CD73. (D) Injected GFP cells (left) in SC tumors express the lung specific marker SP-C (right). (E) Detection of the GFP fluorescence on isolated tumours from subcutaneous injection of lung stem/progenitor cells expressing K-RasG12 with or without p38 activity. (F) Lung tumours from tail-vein injected GFP expressing cells. Each genotype, n = 5. One-Way ANOVA test p≤0.01.
(PDF)
