# Supplementary figures and images for: p38α Negatively Regulates Survival and Malignant Selection of Transformed Bronchioalveolar Stem Cells
Source: PLoS One. 2013 Nov 12;8(11):e78911. doi: 10.1371/journal.pone.0078911 (PMC3827089; doi:10.1371/journal.pone.0078911)

Suppl. Fig. S1

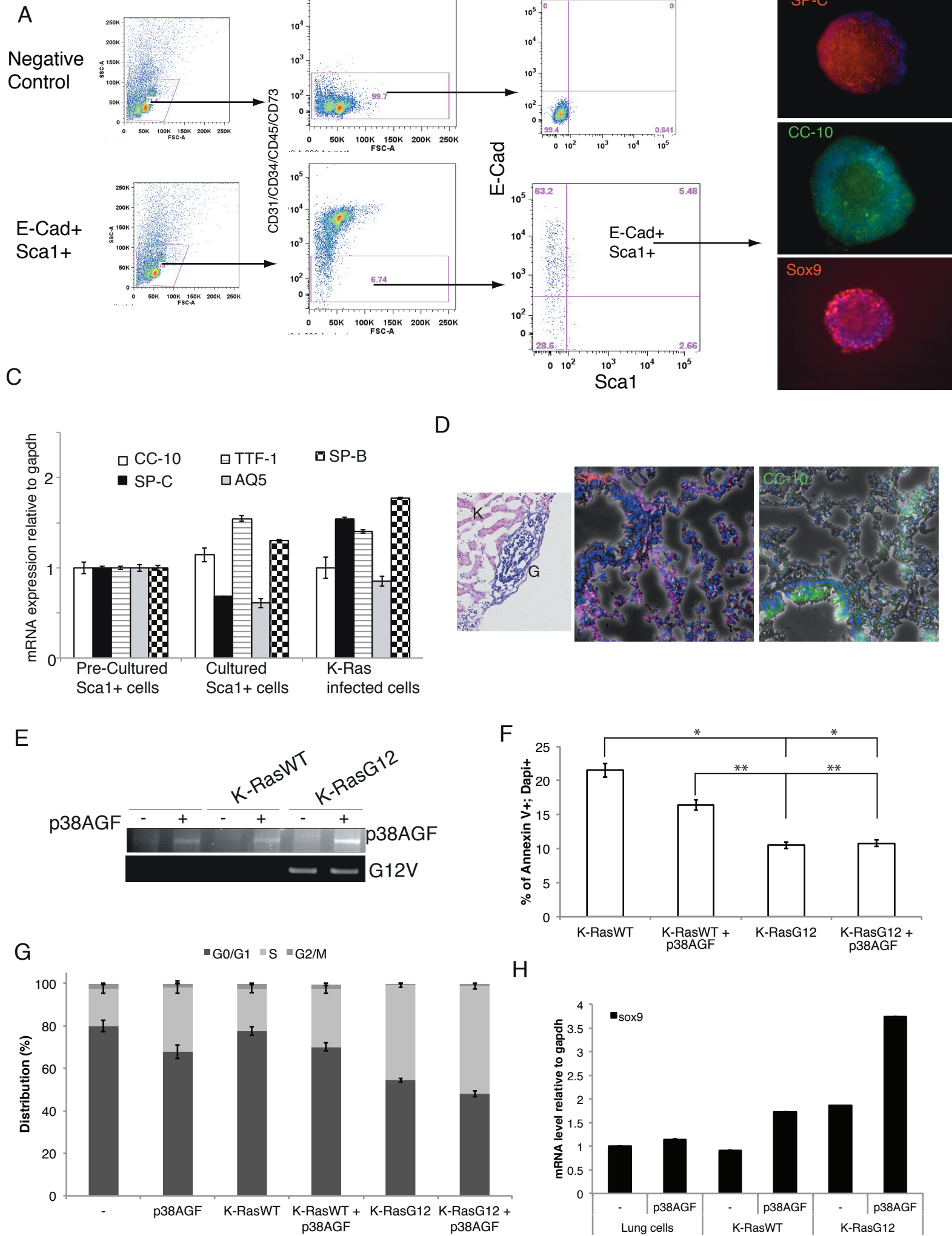

Supplement: Figure S1 — In vitro culture and characterization of K-Ras transformed cells. (A) Immunofluorescence staining of K-Ras12 transformed Sca1 sorted cell spheres in culture showing the expression of epithelial, stem and lung specific markers. (B) Graph shows qPCR relative mRNA expression of lung specific markers in freshly isolated, cultured (passage 30) or K-RasG12 infected (passage 4). (C) Lung stem cells transfected with retrovial vectors expressing K-RasWT or K-RasG12V. The PCR shows the expression of p38AGF (dominant negative), or oncogenic K-RasG12V. (D) Apoptosis of the cell types based on Anexin detection by flow cytometry. (E) Cell cycle distribution of the different cell types. (F) Relative mRNA expression of Sox9 in cells with or without p38 activity. (PDF) [file pone.0078911.s001.pdf]

A

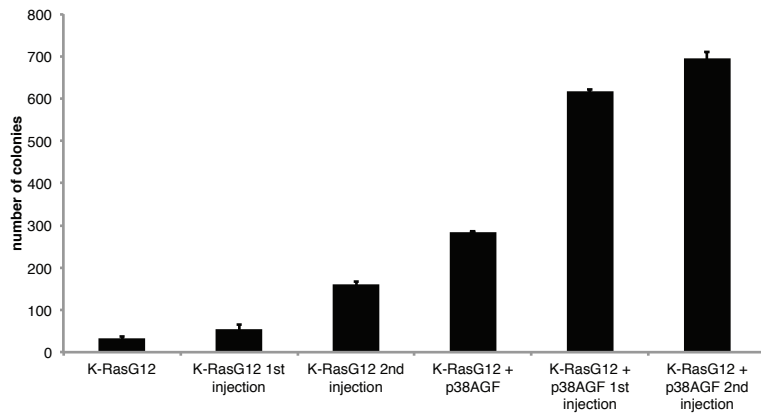

B

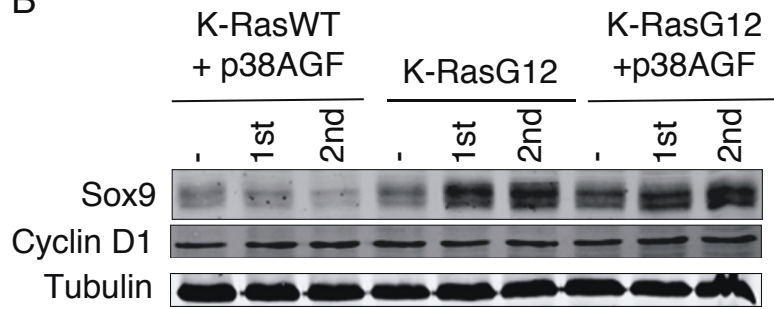

D

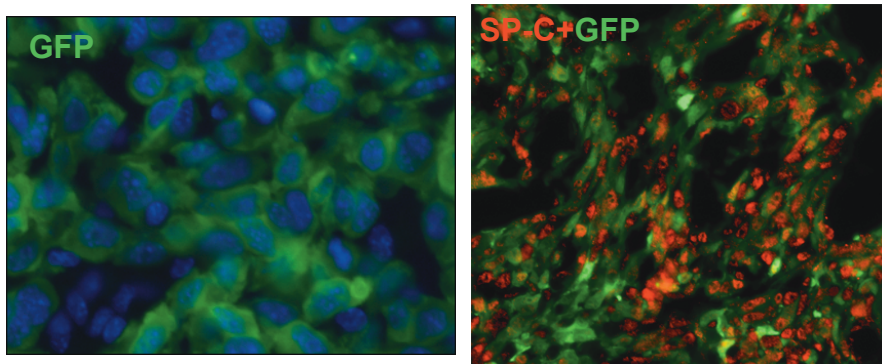

C

Mouse Fibroblasts

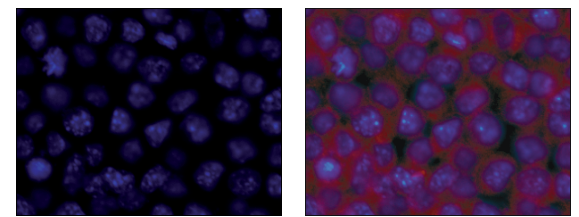

K-RasG12 + p38AGF - 1st Injection

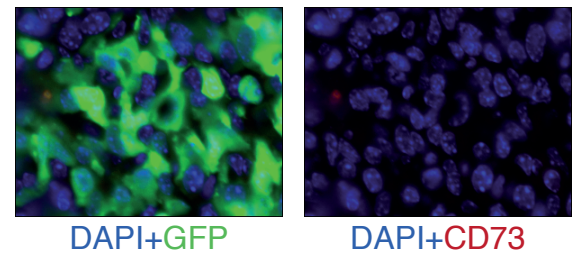

E

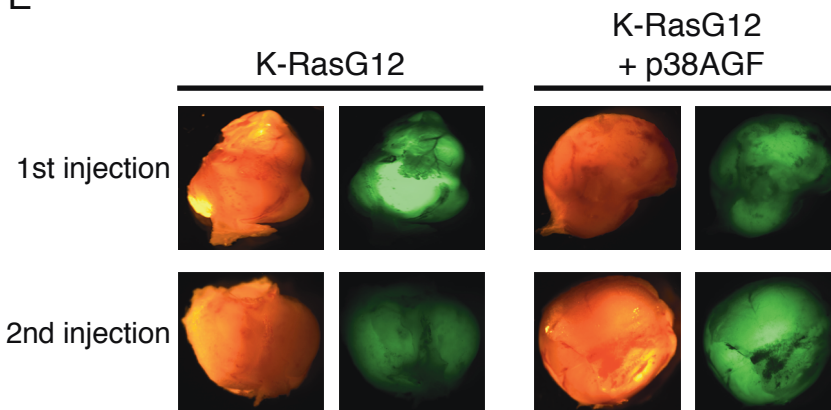

F

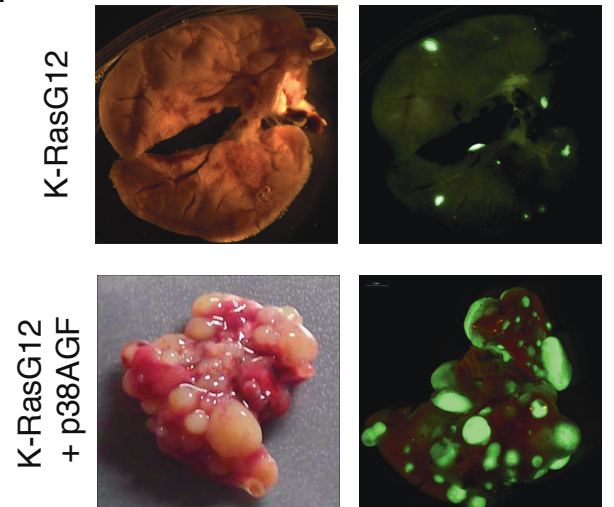

Supplement: Figure S2 — In vitro and in vivo characterization of the functional and molecular properties of the transformed stem cells. (A) Colony formation in soft agar using K-Ras transformed lung stem cells in culture or isolated from serial subcutaneous tumours. Values are depicted as mean ± standard error of the mean (SEM) from four different experiments. (B) Sox9 and Cyclin D1 protein expression in K-RasWT or K-RasG12 with or without p38 activity in culture or cells isolated from serial tumours. (C) Injected GFP cells in SC tumors do not express the non-epithelial marker CD73. (D) Injected GFP cells (left) in SC tumors express the lung specific marker SP-C (right). (E) Detection of the GFP fluorescence on isolated tumours from subcutaneous injection of lung stem/progenitor cells expressing K-RasG12 with or without p38 activity. (F) Lung tumours from tail-vein injected GFP expressing cells. Each genotype, n = 5. One-Way ANOVA test p≤0.01. (PDF) [file pone.0078911.s002.pdf]
